# Autophagy in Premature Senescent Cells Is Activated via AMPK Pathway

**DOI:** 10.3390/ijms13033563

**Published:** 2012-03-16

**Authors:** Liujing Guo, Bushan Xie, Zebin Mao

**Affiliations:** 1 Department of Biochemistry and Molecular Biology, Peking University Health Science Center, 38 Xueyuan Road, Beijing 100191, China; E-Mails: prb@bjmu.edu.cn; 2 Department of Gastroenterology, the First Affiliated Hospital of Nanchang University, Nanchang 330006, China; E-Mail: xiebushan@gmail.com

**Keywords:** autophagy, senescent cells, apoptosis, FoxO3A, AMPK, fibroblasts

## Abstract

Autophagy is a highly regulated intracellular process involved in the turnover of most cellular constituents and in the maintenance of cellular homeostasis. In this study, we show that the activity of autophagy increases in H_2_O_2_ or RasV12-induced senescent fibroblasts. Inhibiting autophagy promotes cell apoptosis in senescent cells, suggesting that autophagy activation plays a cytoprotective role. Furthermore, our data indicate that the increase of autophagy in senescent cells is linked to the activation of transcription factor FoxO3A, which blocks ATP generation by transcriptionally up-regulating the expression of PDK4, an inhibitor of pyruvate dehydrogenase complex, thus leading to AMPK activation and mTOR inhibition. These findings suggest a novel mechanism by which FoxO3A factors can activate autophagy via metabolic alteration.

## 1. Introduction

Premature senescence occurs when cells are exposed to an oxidative or genotoxic stress that causes DNA damage. It also can be induced by an oncogenic stress (such as ectopic overexpression of Ras or Raf by gene transfection) that results in aberrant regulation of cell proliferation. Many types of normal and tumor cells undergo premature senescence after exposure to H_2_O_2_, radiation, or a DNA damaging agent [1–3]. Premature senescent cells exhibit some of the same characteristics as replicative senescent cells, including permanent cell cycle arrest, enlarged and flattened cell morphology, and increased senescence-associated β-galactosidase (SA-β-gal) activity [1,2]. It has been suggested that senescence response, similar to apoptosis, is critical for suppressing tumorigenesis in complex eukaryotes [4,5]. Thus, genetically engineered mice comprised of cells that cannot undergo a normal senescence response typically die prematurely of cancer [6,7]. In contrast to apoptosis, however, cellular senescence does not eliminate cells that are dysfunctional, damaged or potentially neoplastic. Rather, the senescence response irreversibly arrests the proliferation of such cells. Thus, cellular senescence renders cells incapable of forming a tumor (by permanently arresting cell growth), but senescent cells may persist in tissues.

Macroautophagy (hereafter referred to as autophagy) is one of the major pathways for degradation of cellular components in eukaryotic cells that controls the turnover of long-lived proteins and organelles [8]. This evolutionary conserved process is characterized by the formation of double-membrane cytosolic vesicles, called autophagosomes, which sequester cytoplasmic content and deliver it to lysosomes [9]. Although initially identified as a process induced by cellular starvation, an autophagic pathway is now recognized as the cellular response to a variety of stimuli including starvation, hormone treatment, virus infection and various stress conditions [10–12]. To date, functions of autophagy are not well understood. In most circumstances, autophagy appears to promote cell survival by adapting cells to stress conditions. However, autophagy has also been considered as a non-apoptotic program of cell death that is referred to as “autophagic” or “type-II” cell death. In some cellular settings, autophagy is reportedly conducive to cell death when apoptosis is inhibited, acting as a backup mechanism to execute the death process [13].

Autophagy activity has been shown to increase in senescent fibroblasts [14,15]; however, its function and regulation in senescent cells are still unclear. In this study, we used H_2_O_2_-induced senescent cells as a senescent model to investigate the role of autophagy activation in senescent cells. Our results show that autophagy is required for H_2_O_2_-induced senescence process, and inhibition of autophagy may result in the increase of apoptosis during cellular senescent process. These results indicate that autophagy plays a cyto-protective role in senescent cells. Furthermore, we show that FoxO3A activation contributes to the increase of autophagy activity in senescent cells via blocking ATP product.

## 2. Results

### 2.1. Autophagy Activity Increases in Stress-Induced Premature Senescent Cells

**Figure 1:**
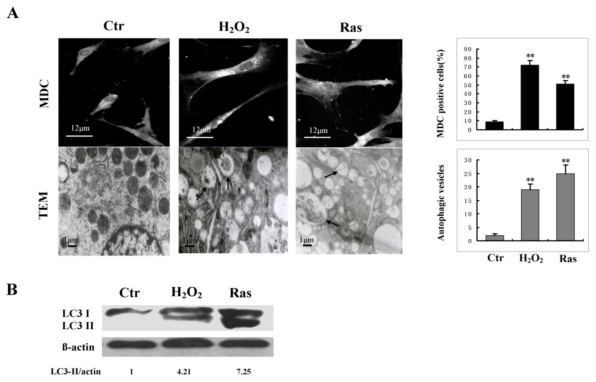
The activity of autophagy increased in senescent cells. (**A**) Morphological analysis of autophagy and cellular senescence in 2BS cells. Upper panel: H_2_O_2_ or RasV12-induced senescent cells were stained with MDC. Young 2BS cells were used as a control. MDC-positive cells (%) were presented as the mean ± SD from three independent experiments. Lower panel: Young and senescent cells were subjected to transmission electron microscopy (TEM). The arrows indicated typical autophagosomes. The number of autophagosome vesicles were counted under one TEM field per cell type, which were presented as the mean ± SD from three independent experiments. Statistical significance was analyzed by using one-way ANOVA and Student-Newman-Keuls analysis. ** indicated a significant difference between senescent and control cells (*P* < 0.01). (**B**) Semiquantitative analysis of the expression of LC3 by immunoblotting. Densitometry was performed for quantification (LC3-II/actin).

To determine whether autophagy was up-regulated in senescent cells, hallmarks of cellular autophagosomes were analyzed in morphological experiments in senescent cells, induced with either sub-lethal concentration of H_2_O_2_ or constitutively active RasV12. Changes of autophagosome morphology were first determined with MDC staining assay (Figure 1A, upper panel). Result showed that the number of MDC-stained punctuate structures, indicative of the formation of autophagosomes [16], was increased in both H_2_O_2_ and RasV12-induced senescent cells. Furthermore, the autophagic process was monitored using transmission electron microscopy (TEM), one of the most reliable measurements for autophagy [17]. It was observed under TEM that the number of double membraned vesicles (characteristics of autophagosome formation) in H_2_O_2_ and RasV12-induced senescent cells was significantly higher than that in young cells. The data indicated that autophagosomes were rarely observed in young cells, but could be readily observed in H_2_O_2_ and RasV12-induced senescent cells (Figure 1A, Lower panel). Altogether, the data from fluorescence and electron microscopy indicated that H_2_O_2_ or RasV12-induced senescence was accompanied by elevated autophagy activity.

To further confirm the observations, immunoblotting assay was carried out to examine the protein expression of LC3, a specific marker for autophagy. Indeed, H_2_O_2_ or RasV12-induced senescent cells had a higher LC3 protein expression as compared with young cells (Figure 1B). Taken together, our results confirmed that autophagy activity was elevated in senescent cells.

### 2.2. Blockage of Autophagy Results in Increased Apoptosis and Impaired Senescence Manifestation During H_2_O_2_-Induced Senescent Process

**Figure 2:**
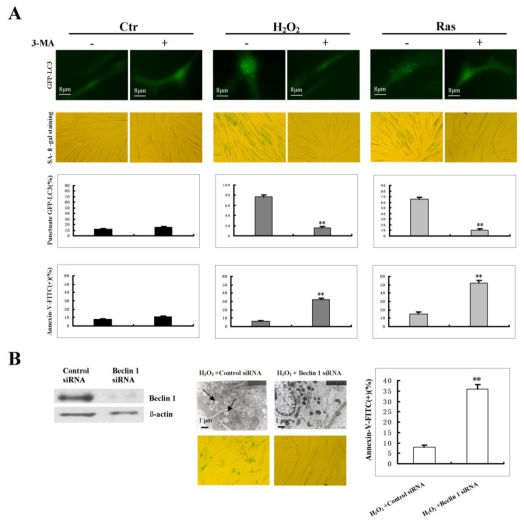
Inhibition of autophagy sensitized cells to oxidative stress. (**A**) First line: inhibition of autophagy with 3-MA was assessed by quantifying the percentage of GFP-LC3 punctuate structure under fluorescence microscope. GFP fluorescence was examined under a Zeiss fluorescence microscope. Second line: cellular senescence was detected using SA-β-gal assay. Third line: quantification of GFP aggregates per cell was performed using the “Analyze particles” command in the Image J software. This experiment was done at day 4 after treatment with 200 μM H_2_O_2_ or constitutively active RasV12 in the presence or absence of 1 mM 3-MA. A minimum of 200 cells were scored, and only cells with more than five puncta were considered positive. Fourth line: apoptosis was analyzed by Flow cytometry analysis. The percentages of Annexin-V positive cells were counted. All values were presented as the mean ± S.D. of three independent experiments. ** indicated a significant difference at the level of *P* < 0.01. (**B**) Left panel: young cells were transfected with Beclin 1-specific dsRNA or a scramble dsRNA for 72 h. The efficiency of Beclin 1 knockdown was confirmed by Western Blot. Middle panel: cells with Beclin 1 knockdown were treated with 200 μM H_2_O_2_ for 2 h as described in Materials and Methods. At day 4 after treatment, autophagy was assessed by transmission electron microscopy, the arrows indicated typical autophagosomes. Cellular senescence was detected using SA-β-gal assay. Right panel: apoptosis was analyzed by flow cytometry as done in (A).

Next, we examined the potential effects of autophagy inhibition on H_2_O_2_ or RasV12-induced senescent process. Young cells were treated with 3-MA (1 mM) to inhibit the activity of class III phosphatidylinositol 3′-kinase, to prevent the formation of autophagosomes and to block autophagy [18]. Results showed that autophagy was evidently inhibited at day 4 after H_2_O_2_ treatment in the presence of 3-MA, which was assessed by the number of punctuate GFP-LC3 (Figure 2A, third line). Subsequently, we employed flow cytometry to examine apoptosis at day 4 after H_2_O_2_ treatment where autophagy was inhibited by 3-MA. Four-day treatment was chosen as the observation point due to the fact that at this time point the premature senescence-induction was very evident in response to 200 μM H_2_O_2_ as described in Materials and Methods. Cells containing Annexin-V-FITC were considered apoptotic. As shown in Figure 2A (fourth line), 3-MA treatment significantly increased apoptosis in H_2_O_2_-treated cells compared to control cells, suggesting that inhibition of autophagy promoted cell apoptosis when cells were exposed to sublethal H_2_O_2_ level. To investigate the relationship between autophagy and senescence, SA-β-gal assay was employed to detect senescence in 3MA treated and untreated cells, it was shown that autophagy was closely linked with senescence since no sign of SA-β-gal positive cells were shown when autophagy was blocked (Figure 2A, second line). Similar result was obtained when senescence was induced by RasV12 in the presence of 3-MA.

To further confirm the observation that inhibition of autophagy induced apoptosis in senescent cells, RNA interference was used to deplete Beclin 1 protein, a molecule that is known to be necessary for autophagy and to regulate autophagic initiation by forming a complex with the class III phosphatidylinositol 3′-kinase [19,20]. The amount of Beclin 1 protein was reduced by at least 90% by the specific siRNA duplex as compared to control siRNA (Figure 2B, left panel). Consistent with previous reports, the reduction of Beclin 1 protein suppressed the formation of autophagosomes, as demonstrated by transmission electron microscopy [13,21] thus blocked the premature senescence process induced by H_2_O_2_ (Figure 2B, middle panel). Subsequent analysis of apoptosis by flow cytometry showed that Beclin 1 knockdown evidently increased the number of apoptotic cells in H_2_O_2_-treated cells as compared to that in control cells (Figure 2B, right panel). These data demonstrated that autophagy activation protected cells from apoptosis in response to external stress, which likely was a prerequisite for cell senescence induction, and hindered autophagic activity abolished the induction of premature senescence process under stress conditions.

### 2.3. Increased Autophagy Is Linked to mTOR Inhibition in Senescent Cells

**Figure 3:**
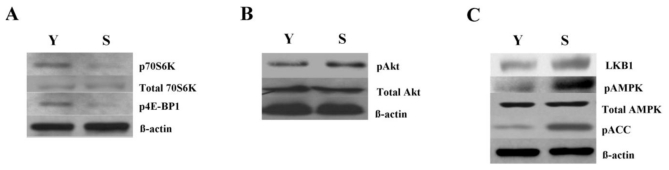
Autophagy increase in senescent cells is linked to the change of AMPK-mTOR activity. (**A**) Immunoblotting analysis of two major mTOR substrates, 70S6K and 4E-BP1, in young and H_2_O_2_-induced senescent cells. The protein extracted from the harvested cells was resolved by SDS-PAGE, transferred to PVDF and probed with the indicated antibodies. (**B**) Immunoblotting analysis of phosphorylated Akt and total Akt in young and H_2_O_2_-induced senescent cells. (**C**) Immunoblotting analysis of AMPK in young and H_2_O_2_- induced senescent cells. Y: young cells; S: H_2_O_2_-induced senescent cells.

Inhibition of mTOR (mammalian Target of Rapamycin) is reported as a common event associated with autophagy induction [22]. To clarify the molecular mechanisms underlying the observed autophagy increase in senescent cells, we first analyzed the status of mTOR signaling pathway in senescent cells. mTOR activity was monitored by analyzing the phosphorylation levels of its two main downstream targets: 70S6K and 4E-BP1 [23,24]. Phosphorylation of 70S6K at Thr389 has been widely described to reflect mTOR activity [25]. Immunoblotting analysis showed that p70S6K level in H_2_O_2_-induced senescent cells was reduced prominently as compared with young cells (Figure 3A), indicating a down-regulation in the activity of mTOR. To further confirm this observation, we analyzed the phosphorylation status of 4E-BP1. Consistent with the observations on 70S6K phosphorylation, we detected a clear decrease in the phosphorylation of 4E-BP1 at Thr37/46 in H_2_O_2_-induced senescent cells (Figure 3A), thus confirming that mTOR activity is down-regulated in senescent cells. Taken together, these results demonstrated that the elevation of autophagy activity observed in senescent cells was linked to mTOR pathway inhibition.

### 2.4. mTOR Inhibition and Increased Autophagy in Senescent Cells Are Linked to AMPK Activation

We then analyzed the activities of Akt kinase and AMPK kinase, two major kinases controlling mTOR activity: The Akt kinase mediates the activation of mTOR in response to growth factors, and the AMPK kinase is involved in mTOR activity inhibition in response to nutritional, genotoxic and metabolic stress signals [26,27]. We first examined the Akt activity in senescent and control cells. Phosphorylation of Akt at Ser473 is often used as an indicator of Akt activity [28]. We found a minimal elevation of Akt phosphorylation at Thr308 in H_2_O_2_-induced senescent cells as compared with control cells (Figure 3B). This suggested that the mTOR pathway inhibition observed in H_2_O_2_-induced senescent cells is not a consequence of a decrease in the Akt activity. Then, we analyzed the AMPK activity to test whether a possible alteration in this kinase could account for the mTOR inhibition observed in senescent cells. For that purpose, we first examined the phosphorylation level of AMPK at Thr172, which has been widely used to monitor AMPK activity [29]. As shown in Figure 3C, AMPK phosphorylation was significantly higher in senescent cells than in control cells. Consequently, phosphorylation of ACC, a substrate of AMPK, was also higher in senescent cells than that in control cells. This indicated an increase of AMPK activity in H_2_O_2_-induced senescent cells. Moreover, we analyzed the total protein content of LKB1, the main kinase involved in AMPK phosphorylation, as the cellular level of this kinase is correlated with AMPK activity [30]. As shown in Figure 3C, immunoblotting analysis revealed an appreciable change in the protein levels of LKB1 in senescent cells as compared with control cells. Taken together, these results suggested that the enhanced autophagy and mTOR inhibition in H_2_O_2_-induced senescent cells were linked to an up-regulation of AMPK activity but not to a decrease in Akt activity.

### 2.5. AMPK Activation Is Linked to the Increase of AMP: ATP Ratio in Senescent Cells

**Figure 4:**
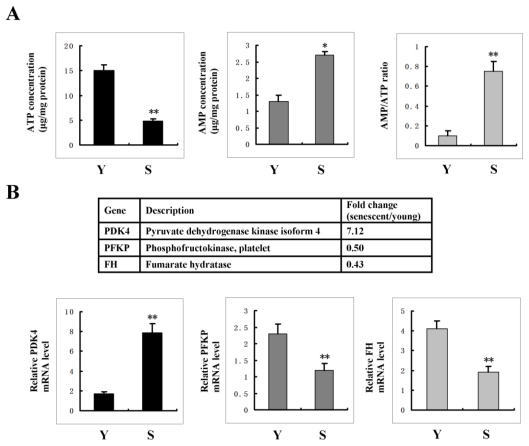
AMPK activation is linked to the increase of AMP/ATP ratio in senescent cells. (**A**) ATP and AMP levels in senescent and young cells were quantified by reverse-phase HPLC. Data represented as mean ± S.D. of three independent determinations. * indicated a significant difference at the level of *P* < 0.05, ** indicated a significant difference at the level of *P* < 0.01. (**B**) Upper panel: changes of the expression of three energy production-related genes, PDK4, PFKP and FH were analyzed with microarray in senescent cells. Lower panel: change of the expression of the three genes was confirmed by real-time PCR. Data represented as mean ± SD of three independent experiments. Y: young cells; S: senescent cells. ** indicated a significant difference at the level of *P* < 0.01.

To clarify the molecular basis underlying AMPK activation in senescent cells, we first analyze cellular AMP: ATP ratio. As shown in Figure 4A, senescent cells displayed an increased level of AMP with a decreased level of ATP. Thus, the ratio of AMP/ATP greatly increased in senescent cells as compared with control cells, thereby suggesting that AMPK activation was linked to the increase of AMP/ATP ratio in senescent cells.

Next, we analyzed the transcriptional profiles of genes involved in energy production with mRNA microarray analysis to test whether there was an increase of AMP/ATP ratio resulted from the impaired energy generation. Comparing senescent cells with control cells, three genes, PDK4, PFKP and FH, were expressed at different levels. PDK4 is located in the matrix of the mitochondria and inhibits the pyruvate dehydrogenase complex by phosphorylating one of its subunits [31], thereby inhibits the transition (decarboxylation) of glycolysis end product pyruvate to mitochondrial acetyl-CoA, with net result of curtailing mitochondrial glucose oxidation. PFKP is a key regulatory enzyme in glycolysis, it catalyzes the irreversible conversion of fructose-6-phosphate to fructose-1,6-bisphosphate. FH is an enzymatic component of the tricarboxylic acid cycle; it catalyzes the formation of l-malate from fumarate. Among these three genes, PDK4 expression increased in senescent cells as compared with that in control cells, whereas PFKP and FH expressions decreased in senescent cells (Figure 4B). The altered expression of these enzymes in senescent cells caused the blockage of glycolysis, subsequent reduction of ATP production and increase of AMP: ATP ratio in senescent cells.

### 2.6. FoxO3A Activation Transcriptionally Up-Regulates PDK4 Expression and Contributes to the Increase of AMP: ATP Ratio in Senescent Cells

**Figure 5:**
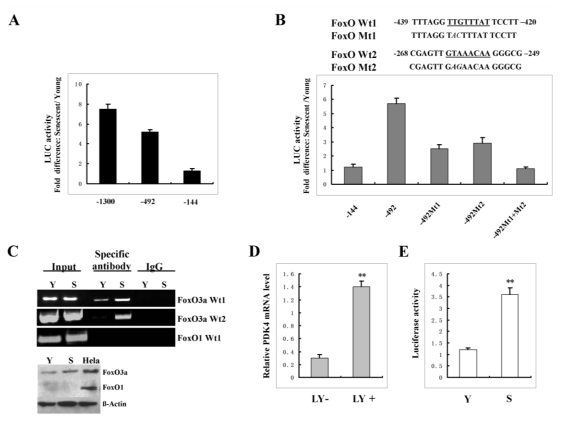
FoxO3A transcriptionally up-regulated PDK4 expression in senescent cells. (**A**) 5′-deletion analysis of the PDK4 promoter. Various deletion constructs were transiently co-transfected with pRL-CMV (as an internal control) into young and senescent cells (left panel). The bar graph in the right panel represented the fold difference in luciferase (LUC) activity in senescent versus young cells after normalizing for pRL-CMV activity. The results represented luciferase activity from three independent transfections. The numbers represented the positions relative to the transcriptional starting site of the PDK4 gene. (**B**) Mutations of two FoxO binding sites attenuated the fold difference in luciferase (LUC) activity in senescent versus young cells. (**C**) Upper panel: ChIP analysis of *in vivo* FoxO binding in senescent and young cells. Chromatin immunoprecipitation was performed with the indicated antibody, followed by PCR of the PDK4 promoter region containing the consensus FoxO binding site as indicated in (B). Lower panel: Western Blot analysis of FoxO3A and FoxO1 expression in 2BS fibroblasts. Y: young cells; S: senescent cells. (**D**) RNA was prepared at 48 h after LY294002 treatment and real-time PCR was used to analyze PDK4 mRNA level. The values were represented as the Mean ± SD of three independent experiments. (**E**) Young or senescent cells were transiently transfected with a synthetic promoter containing three FoxO3A response elements (FHRE). Luciferase activity was measured 36 h post-transfection. The experiment shown is a representative experiment of a minimum of three independent experiments.

PDK4 promoter was cloned and analyzed for potential regulatory regions that may be responsible for the up-regulation of PDK4 detected in senescent cells. To determine the basal activity of PDK4 promoter in young and senescent cells, a 1.3-kb PDK4 promoter fused to the luciferase reporter gene was used in transient transfection studies. Various pPDK4 5′-deletion constructs were generated (Figure 5A) and transiently co-transfected with a cytomegalovirus-driven *Renilla* luciferase vector (as an internal control) into young and senescent cells. After 36h of incubation, cells were harvested and analyzed for luciferase activity. As shown in Figure 5A, luciferase activity induced by the full-length PDK4 promoter was approximately 7-fold greater in senescent cells than that in young cells. Deletion of segments from the 5′ end, from −1300 to −492, slightly reduced the difference in expression between young and senescent cells. However, further deletion to −144 upstream the transcription starting site of the PDK4 promoter greatly reduced the difference in luciferase activity. These data indicated that the increased expression of PDK4 in senescent cells required the promoter region from −492 to −144.

To determine the specific transcription factor binding elements within this region, we performed a detailed computer analysis using the MatInspector program and found two FoxO transcription factor binding elements. Two base mutations within each FoxO element were introduced by site-directed mutagenesis in the −492 construct (Figure 5B). We transfected the promoter −492 construct and the mutated constructs into young and senescent cells respectively and measured the luciferase activity from each construct after normalizing for pRL-CMV control. As shown in Figure 5B, mutation of each FoxO element lessened the difference in luciferase activity; mutations of two FoxO elements completely eliminated the difference between senescent and young cells. These data suggested that FoxO transcription factor contributed to the elevation of PDK4 gene expression in senescent cells.

Next, ChIP assay was performed to determine whether FoxO could bind to and activate transcription from the endogenous PDK4 promoter. Interaction between FoxO and the PDK4 promoter region that contains the two FoxO binding sites was detected by a specific PCR assay of immunoprecipitated FoxO-DNA complexes. Protein-DNA complexes were cross-linked and DNA sheared as described in Materials and Methods. FoxO1 antibody was used to immunoprecipitate DNA since it has been shown that FoxO1 can regulate PDK4 expression [32]. Results show that no PCR band was detected whether in young cells or in senescent cells. However, PCR product was observed in senescent cells but not in young cells when FoxO3A antibody was used (Figure 5C). These results showed that FoxO3A, but not FoxO1, can bind specifically to the endogenous PDK4 promoter. Further analysis showed that no FoxO1 expression was detected in 2BS fibroblasts (Figure 5C, Lower panel). This finding could explain the observation that no FoxO1 bound to the endogenous PDK4 promoter. Instead, a high level of FoxO3A expression was observed in this type of cells (Figure 5C, Lower panel), suggesting that FoxO3A binding activated PDK4 gene expression in senescent cells. To further confirm that FoxO3A up-regulates PDK4 expression, a PI3K-Akt pathway inihibitor, LY294002, was employed. Inhibition of PI3K-Akt results in the up-regulation of FoxO3A activity. As shown in Figure 5D, LY294002 treatment of young cells dramatically increased PDK4 expression. Furthermore, data showed that FoxO3A activity increased in senescent cells compared to young cells (Figure 5E). Taken together, our results indicated that FoxO3A was required for the up-regulation of PDK4 expression in senescent cells.

### 2.7. Constitutively Active FoxO3A Up-Regulates Autophagy Activity in Young Cells

**Figure 6:**
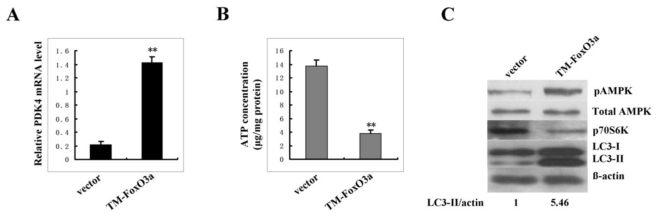
FoxO3A activation up-regulates autophagy activity in young cells. FoxO3A-pBABE and empty pBABE vector were packaged in pheonix cells for 48 h, supernatants were collected to infect young 2BS cells. 2BS cells were then maintained in puromycin at a concentration of 0.5 μg/mL for 7 days. (**A**) Real-time PCR detection of PDK4 mRNA level in FoxO3A overexpression clones and control clones. (**B**) Reverse-phase HPLC analysis of ATP concentration in FoxO3A overexpression clones and control clones. (**C**) Total and phosphorylated AMPK, 70S6K phosphorylation and LC3 protein level by western blot analysis in FoxO3A overexpression clones and control clones.

Next, we test whether FoxO3A activation up-regulates autophagy activity. Young cells were infected with pBabe retrovirus expressing constitutively active FoxO3A, which is mutated to alanine at all three phosphorylation sites for Akt. Results showed that constitutive active FoxO3A induced PDK4 basal expression up to 6-fold (Figure 6A). Importantly, expression of active FoxO3A resulted in the decreased ATP level (Figure 6B), the increased AMPK activity, the decreased mTOR activity and the increased autophagy activity (Figure 6C). It is worth noting that the increase of AMPK activity is not a consequence of the up-regulation of LKB1 expression as FoxO3A activation did not change LKB1 level. These data indicate that FoxO3A may up-regulate autophagy activity via blocking ATP production.

### 2.8. Knockdown of FoxO3A Inhibits Autophagy Activity in Senescent Process

**Figure 7:**
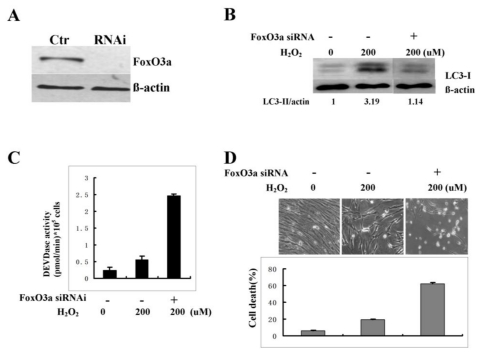
Knockdown of FoxO3A inhibits autophagy activity in senescent process. (**A**) Confirmation of FoxO3A knockdown by RNA interference in young cells. (**B**) Semiquantitative analysis of the expression of LC3 by immunoblotting four days after H_2_O_2_ treatment. Densitometry was performed for quantification (LC3-II/actin). (**C**) DEVDase activity was measured four days after H_2_O_2_ treatment. (**D**) Cell morphology four days after H_2_O_2_ treatment and cell death was determined by Trypan blue exclusion test. Data were expressed as the percentage of dead cells relative to total cells. The values are represented as the mean ± SD of three independent experiments.

Finally, we tested whether the knockdown of FoxO3A inhibits autophagy activation in senescent process. For this purpose, RNA interference was used to deplete FoxO3A protein. The amount of FoxO3A protein in the cells was reduced by at least 90% by the specific siRNA duplex as compared to control siRNA (Figure 7A). Subsequently, cells with FoxO3A or control siRNA treatment were induced to senescence by H_2_O_2._ Four days after H_2_O_2_ exposure, autophagy activity was assayed by examining LC-3 protein expression. As shown in Figure 7B, FoxO3A siRNA treatment inhibited autophagy activity compared with control. Consistent with the cytoprotective function of autophagy in senescent cells, the knockdown of FoxO3A promoted cell apoptosis in senescent process as judged by caspase-3 activity (Figure 7C) and the number of cell death (Figure 7D). These data indicate that FoxO3A is required for autophagy activation in senescent process.

## 3. Discussion

Our results demonstrated that autophagy plays a cyto-protective role in senescent cells. This is supported by following observations: autophagy activity increased in senescent cells compared to young cells; inhibiting autophagy caused the increase of cell death and hindered senescence process in the course of senescence induction; the cyto-protective role of autophagy is consistent with its effect on lifespan extension.

Transcription factor FoxO has been shown to regulate autophagy activity, but the mechanism is poorly understood. Several reports demonstrated that FoxO negatively controls mTOR signaling in various organisms. For example, in C.elegans Daf-16 (FoxO) negatively regulates the expression of Daf-15 (Raptor) [33], and in skeletal muscle FoxO1 down-regulates mTOR, Raptor and other components of the mTOR signaling pathway [34,35]. In addition, recent two reports showed that in muscle atrophy, FoxO3A activates autophagy via transcriptionally regulating the expression of many autophagy-related genes, including LC3B, Bnip3 and Bnip3L [36,37]. In the present study, we found that FoxO3A regulated autophagy activity through a new pathway in senescent cells. FoxO3A activation in senescent cells inhibits glucose catabolism by transcriptionally up-regulating the expression of PDK4, blocking ATP generation, hence causing the AMPK activation and mTOR inhibition that results in the increase of autophagy activity in senescent cells. These findings suggested a novel mechanism by which FoxO3A factors can activate autophagy.

FoxO3A activity is regulated primarily by post-translational modifications, including phosphorylation, acetylation and ubiquitination, which depend on environmental stimuli [38]. In mitogenic conditions, Akt is a primary regulator of FoxO3A. Upon activation following mitogenic stimulation, Akt translocates to the nucleus and phosphorylates FoxO3A, decreasing FoxO3A activity. Data from our and other studies showed that Akt activity increased in H_2_O_2_-induced senescent cells [39]; however, we found that FoxO3A activity did not decrease but did increase in H_2_O_2_-induced senescent cells. There are two possible explanations for the inconsistency between Akt activity and FoxO3A activity: (1) the increase of FoxO3A activity is likely resulted from the change of other post-translational modification; (2) nuclear translocation of Akt is impaired in H_2_O_2_-induced senescent cells.

The increase of FoxO3A activity is also observed in Raf-induced senescent cells and replicative senescent cells [40,41], but the significance of FoxO3A activation in senescent cells is unknown. It has been shown that FoxO3A contributes to age-related decline in tissue function by increasing proteolysis of matrix components via up-regulation of collagenase expression [41]. In this study, we showed that FoxO3A activation protects cells from apoptosis in senescence process; this is achieved, at least partly, by up-regulating autophagy activity. It has been well known that activated FoxO protein may promote stress resistance by binding to the promoter of the genes encoding manganese superoxide dismutase (MnSOD) and catalase, two scavenger proteins that play essential roles in oxidative detoxification in mammals [42]. Therefore, it cannot be ruled out that this pathway contributes to the cyto-protective function of FoxO3A in senescence process.

Taken together, our results indicated that the autophagy activity increased in senescent cells. Inhibiting autophagy may cause the increase of cell death, suggesting that autophagy activation plays a protective role in senescent cells. Furthermore, analysis of the mechanism underlying the increase of autophagy activity in senescent cells showed that FoxO3A contributes to AMPK activation and mTOR inhibition through blocking glucose catabolism and subsequent ATP production. However, it cannot be ruled out that other pathways are involved in the increase of autophagy activity in senescent cells.

## 4. Materials and Methods

### 4.1. Materials

3-methyladenine (3-MA) and monodansylcadaverine (MDC) were purchased from Sigma Chemical Co. (St. Louis, MO, USA). Antibody against microtubule-associated protein 1 light chain 3 (LC-3) was also purchased from Sigma. Antibodies against p70S6K (Thr389), p4E-BP1 (Thr37/46), pAkt (Thr308), pTSC2 (Thr1462), pAMPK-α (Thr172), LKB1 and pACC (Ser79) were purchased from Cell Signaling Technology, Inc. (Beverly, MA, USA). Antibodies against Beclin 1 and β-actin were obtained from Santa Cruz Biotechnology (Santa Cruz, CA, USA).

### 4.2. Cell Culture

Human embryonic lung diploid fibroblast 2BS cells (obtained from the National Institute of Biological Products, Beijing, China) were previously isolated from female fetal lung fibroblast tissue and have been fully characterized [11]. The cells were maintained in Dulbecco’s modified Eagle’s medium (Invitrogen, Carlsbad, CA, USA) supplemented with 10% (v/v) fetal bovine serum (Invitrogen), glutamine (2 mM; Invitrogen) and penicillin/streptomycin (100 units/mL/100 μg/mL; Invitrogen) at 37 °C in 5% CO_2_. Cells at early passages (between 16 and 25 passages) were used in all experiments to avoid complications of replicative senescence as 2BS cells have a mean life span about 55–70 passages. For the induction of premature senescence, 2BS cells at about 75% confluence were briefly exposed to 200 μM H_2_O_2_ (diluted in Dulbecco’s Modified Eagle’s medium supplemented with 10% FBS) for 2 h. The cells were washed twice with Dulbecco’s Modified Eagle’s medium (DMEM) to remove H_2_O_2_ and re-cultured in fresh complete medium. Cell senescent phenotype was determined by SA-β-gal staining, cell proliferation and morphology at 3–5 days after H_2_O_2_ treatment.

### 4.3. Labeling of Autophagic Structures with MDC

2BS cells growing on coverslips were incubated with 0.05 mM MDC added directly to the culture medium. After incubation at 37 °C for 1 h, the cells were fixed in 4% paraformaldehyde for 15 min and immediately analyzed using a Zeiss epifluorescence microscope (Germany) with excitation and emission at 380 nm and 525 nm wavelength, respectively.

### 4.4. Electron Microscopy

The cells were harvested by trypsinization, washed twice with PBS and fixed with ice-cold glutaraldehyde (3% in 0.1 M cacodylate buffer, pH 7.4) for 30 min. After washing in PBS the cells were post-fixed in OsO_4_ and embedded in Epon; 0.1 mm thin sections were stained with uranyl acetate/lead citrate (Sigma-Aldrich, St. Louis, MO, USA) and viewed by a JEM1230 electron microscope (Japan).

### 4.5. Immunoblotting

Cells pellets were lysed in Triton X-100/glycerol lysis buffer plus 1% sodium deoxycholate and 0.1% SDS, and then electrophoresed and transferred onto nitrocellulose membranes. After blocking with 5% non-fat dry milk, the membranes were incubated with the primary antibodies at 1:1000 dilution overnight at 4 °C followed by the secondary antibodies conjugated with horseradish peroxidase at 1:5000 dilution for 2 h at room temperature. Protein bands were visualized on X-ray film using an enhanced chemiluminescence system (Pierce Biotechnology), and quantitatively analyzed via densitometry.

### 4.6. Flow Cytometry

The cells were trypsinized, washed with PBS twice, and then adjusted to 5 × 10^5^ cells/500 μL in the binding buffer prior to incubation with Annexin V-fluorescein isothiocyanate (FITC) and propidium iodide (PI) according to the instructions of an Annexin-V-FITC Apoptosis Detection Kit (Biovision Inc, Mountain View CA) and finally analyzed by flow cytometry (FACS Aria, Becton Dickinson). All the floating cells were harvested for apoptotic analysis.

### 4.7. Caspase-3 Activity Assay

Caspase-3 Cellular Activity Assay Kit PLUS (AK-703) (Biomol, Germany) was used. The assay is based on the cleavage of the specific fluorogenic peptide substrate acetyl-aspartyl-glutamyl-valyl-aspartyl- 7-amino-4-trifluoromethylcoumarin (Ac-DEVD-AFC) for caspase-3-like activity. Sample cells were harvested at various time points post-treatment and treated according to the manufacturer’s instruction. Fluorescence of AFC, which was proportional to the DEVD-ase activity in the cell lysates, was recorded on fluoroscan Ascent FL (Labsystems, Finland) at an excitation/emission wavelength of 390/510 nm, respectively.

### 4.8. Microarray Analysis

RNAs of young (22 PD) and senescent (61 PD) fibroblasts were extracted using Trizol Reagent (Invitrogen). The samples (preserved in DEPC water and transferred with dry ice) were then sent to CapitalBio Technology, Co., Ltd. for the detection of genes differently expressed between young and senescent cells. The product No. of gene chip used was 220,010, Jingxin human whole genome oligonucleotide microarray V1.0 with 22,000 probes.

### 4.9. SA-β-gal Staining

For detection of SA-β-gal activity, cells were washed twice with phosphate-buffered saline (PBS, pH 7.2), fixed with 4% formaldehyde for 5 min at 22 °C, and washed with PBS (pH 6.0) again. Cells were then incubated overnight at 37 °C without CO_2_ in freshly prepared staining buffer (1 mg/mL X-gal, 40 mM citric acid/sodium phosphate, pH 6.0, 5 mM potassium ferrocyanide, 5 mM potassium ferricyanide, 150 mM NaCl, 2 mM MgCl_2_).

### 4.10. siRNA Transfection

The cells were seeded at 40% confluence per well in 6 well plates overnight and transfected with Beclin 1 siRNA or FoxO3A siRNA or control siRNA duplex (Santa Cruz Biotechnology, CA, USA) using Lipofectamine 2000. Successful targeted knockdown was verified by Western Blot.

### 4.11. Plasmid Construct

To obtain various deletion constructs of human PDK4 promoter plasmids, a series of forward primers and a common reverse primer were used to amplify the PDK4 promoter fragments by PCR from human genomic DNA. The various forward primers were used: −1300, 5′-AA *CTC GAG*CGA GGT TGC AGT GAG CCG AGA TG-3′; −492, 5′-AA *CTC GAG*TGT GCC GGT CTC TTC TGA AGA AAA G-3′; −144, 5′-AA *CTC GAG*CTT CCC ACC CTT TTT CCG TCA-3′; (the underlined sequence is the Xho I (NEB, Beverly, MA, USA) recognition site). The common reverse primer: +69, 5′-AA *AAG CTT*CCG CCG CCG CCC GAG GTT TTA-3′ (the underlined sequence is the *Hin*dIII (NEB) recognition site). Each pair of primers was used to amplify various sizes of promoter region. The resulting fragments were subjected to restriction digestion and subcloned into pGL3-basic plasmid (Promega, Madison, WI, USA). These clones were designated as −1300 (full-length), −492, −144 relative to the transcriptional start site.

### 4.12. Transient Transfection and Luciferase Assay

For PDK4 promoter luciferase assay, pGL3-PDK4 and pRL-CMV (Promega) were transiently transfected into 2BS cells using Lipofectamine 2000 transfection reagent. After 36 h of incubation, cells were lysed in passive lysis buffer (Promega), and luciferase activity was measured using Luciferase Assay System (Promega) with a luminometer (Lumat LB 9501; Berthold).

### 4.13. Site-Directed Mutagenesis

Bases were mutated using the QuikChange Site-Directed Mutagenesis Kit (Stratagene, La Jolla, California, USA). Two promoter mutagenic primers were used (primer1 for FoxO binding site 1: 5′-CGC AGG TCT TTA GGT *AC*T TTA TTC CTT TCT CT-3′; primer2 for FoxO binding site 2: 5′-ACA GCC TCC GAG TTG *AG*A ACA AGG GCG AGC CT-3′. The underlined bases are the mutated bases) were synthesized. Pfu Turbo DNA polymerase was used to synthesize the mutagenic promoter and 3′UTR, followed by digestion of the parental plasmids by *Dpn*I according to the manufacturer’s instructions. The mutated plasmid was transformed into XL1-Blue competent cells and the resulting plasmid was isolated and sequenced to confirm the mutations.

### 4.14. Chromatin Immunoprecipitation

Chromatin immunoprecipitations were performed using the Chromatin Immunoprecipitation Assay Kit (Upstate). Antibodies used for ChIP were anti- FoxO3A. Primer pairs used for PCR were as following: FoxO binding site 1 (−439 to −420), forward primer 5′-GAA GAA AAG GGG GCG GTG GG-3′ and reverse primer 5′-GCC AAC AAG CTG CGT TCA AAA GT-3′. FoxO binding site 2 (−268 to −249), forward primer 5′-CTC TGA GCA AGG ACC AAT GAG CA-3′ and reverse primer 5′-TGG GGC AAC GTG GGC TTA AGA TT-3′.

### 4.15. Nucleotide Extraction and AMP/ATP Analysis

Cells were washed twice with ice-cold PBS. Thereafter, cells were harvested by trypsinization followed by centrifugation at 3000 g for 10 min at 4 °C. Cell pellets were then washed in ice-cold DMEM and centrifugated at 10,000 g for 10 min. Cells were lysed by the addition of trichloroacetic acid (0.73 M, final concentration), vortexed and then incubated on ice for 2 min. The resulting suspension was cleared by centrifugation at 15,000 g for 2 min. An aliquot of the supernatant was neutralized with ice-cold KOH. Total cellular AMP and ATP were quantified by reverse-phase HPLC of trichloroacetic acid extracts using a Vydac C18 reverse-phase column. Protein concentration was determined spectro-photometrically using the BCA protein assay kit.

### 4.16. Real-Time PCR

Total RNA was extracted using Trizol reagent (Invitrogen Corp) according to the manufacturer’s protocol. The first strand cDNA was synthesized with SuperScript III (Invitrogen Corp), and PDK4, PFKP and FH mRNAs were analyzed by real-time PCR with the following primers: PDK4: forward primer 5′-GGT TAT TGT TGT CTT GGG AAA-3′, reverse primer 5′-CTT TGC ATA CAG ACG AGA AAT-3′. PFKP: forward primer 5′-CTT TGG ATG GGC TTG GGA T-3′, reverse primer 5′-TGC TGG GGT TAC AGG TGT G-3′. FH: forward primer 5′-TCA GTG TGA AGC AAT GAC CA-3′, reverse primer 5′-AAC TGA AGC ATC CCC CAG CA-3′). The GAPDH primers were: forward primer 5′-AGA AGG CTG GGG CTC ATT TG-3′ and reverse primer 5′-AGG GGC CAT CCA CAG TCT TC-3′.

### 4.17. Statistical Analysis

All experiments were repeated three times and similar results were obtained. Statistical analysis of the difference between groups was performed using Student’s t-test. Significance was established when *P* < 0.05.

## 5. Conclusions

We have demonstrated that the activity of autophagy increases in H_2_O_2_ or RasV12-induced senescent fibroblasts. Inhibiting autophagy promotes cell apoptosis in senescent cells, suggesting that autophagy activation plays a cytoprotective role. Furthermore, our data indicate that the increase of autophagy in senescent cells is linked to the activation of transcription factor FoxO3A, which blocks ATP generation by transcriptionally up-regulating the expression of PDK4, an inhibitor of pyruvate dehydrogenase complex, thus leading to AMPK activation and mTOR inhibition. These findings suggest a novel mechanism by which FoxO3A factors can activate autophagy via metabolic alteration.
